# Compartmental analysis of retinal vascular parameters and thickness in myopic eyes using SS-OCTA

**DOI:** 10.3389/fmed.2024.1521710

**Published:** 2024-12-20

**Authors:** Chen Zeng, Chong Tang, Yixin Tan, Juxian Liu, Kai Shi, Qi Li

**Affiliations:** Chongqing Key Laboratory of Prevention and Treatment on Major Blinding Diseases, Chongqing Eye Institute, Chongqing Branch (Municipality Division) of National Clinical Research Center for Ocular Diseases, The First Affiliated Hospital of Chongqing Medical University, Chongqing, China

**Keywords:** myopia, optical coherence tomography angiography, optic disc, retinal nerve fiber layer (RNFL), retinal vessel density, retinal blood flow, axial length (AL), corneal curvature (CR)

## Abstract

**Background:**

This study aimed to comprehensively explore the thickness and topographic distributions of retinal vessel alterations of different myopic eyes by using swept-source OCT angiography (SS-OCTA).

**Methods:**

One hundred myopes were included in this observational cross-sectional study. All participants underwent a series of ocular examinations of biometrical parameters, including spherical equivalent refraction (SER), axial length (AL), intraocular pressure (IOP), curvature radius (CR), and others. Retinal parameters like vessel density (VD) of different compartments of papillary and peripapillary sectors were measured by SS-OCTA, respectively. Two sample-independent T-test was applied to identify intraocular differences in retinal biometrical indicators between groups, and correlation analysis was used to explore potential relationships between AL/CR ratio and some ocular variables.

**Results:**

For high myopic participants, they exhibited a lower vessel density, a lower small vessel density, and a lower flow area, especially in the superficial layer and the nerve fiber layer (RNFL), along with a thinner superficial layer, RNFL and retina. More alterations were proved in nasal peripapillary sectors in high myopes. We also explored their hidden relationship with AL/CR ratio. We found that in non-high myopes, the thickness of the whole retina, RNFL and the superficial layer were all negatively correlated with AL/CR ratio in the papillary and peripapillary zone. In contrast, the vessel density and flow area of several vessel layers were positively correlated. However, there wasn’t so much significance found in high myopic eyes.

**Conclusion:**

Retinal vessel microstructure was more easily affected in highly myopic eyes, especially in superficial blood vessels, and compartmental analysis showed that alterations in nasal peripapillary sectors were more evident. Additionally, we highlighted hidden correlations between AL/CR ratio and blood flow characteristics of specific vascular layers, which could serve as sensitive biometrical indicators of early retinal damages.

## Introduction

The prevalence of myopia has been one of the highest worldwide while having reached epidemic proportions in East Asia already (1). Based on a meta-analysis collecting the global prevalence of myopia in the last 20 years, it is predictable that 49.8% of the world population is likely to become myopic by 2050 (2), which is similar to another epidemiological study, suggesting myopia will cover half of the world population (3).

OCTA is widely performed as a reliable, noninvasive technique for detecting microcirculation of retinal and choroidal vessels, aiming to provide abundant information on depth-resolved fundus vascular images for myopic patients. Generally, a series of vascular layers such as superficial and deep retinal capillary plexus and choroidal capillaries are quantitatively portrayed by OCTA, as it is also beneficial to describe the impact of myopia on the microvasculature of both macular and papillary regions. Swept-source OCT angiography (SS-OCTA) is originally performed as an alternative detecting method for generating ocular vasculature. Compared to spectral-domain OCT (SD-OCT), swept-source OCT (SS-OCT) is developed to get longer wavelength (1040–1060 nm) with a deeper resolution of fundus vasculature while laying the foundation for SS-OCTA to embrace a better visualization of the chorioretinal vascular images (4). Therefore, SS-OCTA is widely served as an auxiliary tool for the diagnosis of multiple ocular diseases, such as detecting potential glaucomatous damages in high myopia (5), where we identify several focal sectoral capillary dropouts with no visible microvascular network in choroidal layers (6). However, owing to the lack of velocities of particular compartments of fundus vessels, there are still limitations for SS-OCTA to offer more precise quantitative results of chorioretinal vasculature (7).

Changes in several chorioretinal biometrical parameters are pathological results of myopia, especially in high myopia. Recently, visual signaling occurring on the retina has proved to be an imperative process of scleral remodeling, suggesting that variables of retinal vascular parameters may be correlated to the progression of myopia, such as axial elongation (8). Researchers preferred to divide retinal capillaries into the superficial vascular plexus (SVP) and the deep capillary plexus (DCP) and analyze the density and the flow area of different vessel layers. Wang et al. reported that the vessel density of both SVP and DCP are substantially lower in high myopic eyes than those of non-high myopes, which is consistent with the results of Cheng et al., who suggest the vessel density of DCP in the macular region of high myopia reduced as well (9, 10). Further, similar pathological changes of retinal capillary plexus happened to eyes with longer axial lengths of anisometropic children. Researchers suggest it is possibly attributed to the decreasing histological metabolism during axial elongation, leading to a thinner RNFL and the degeneration of retinal microvasculature (11).

On the contrary, there were not so many significant discrepancies when Ye et al. explored the variance of vessel densities of SVP between simple and pathological high myopic patients, though their DCP still exhibited differences (12). Some other studies focused on the changeable retinal thickness of myopic eyes. Since childhood, there has been a prolonged tiny decrease in the topographical thickness distribution of macular retinal thickness, whereas it seemed to get more steady over 18 months of myopia progression (13). After repeated low-level red-light therapy, pediatric retinal thickness was about to be restored just partially, especially in the macular region, while lacking significant impacts on microcirculation (14).

As another main source of retinal microcirculation, the impact on the choroid is also essential when looking into the etiology and pathological effect of myopia. Choriocirculation always comprises three independent vessel layers, defined as the choriocapillaries, Sattler’s layer and Haller’s layer, comprised of different types of fundus vessels. Choroidal thickness (CT) has been widely used as a typical biomarker of fundus damage of myopia. During the increasing severity of myopia, the average CT begins to reduce significantly and constantly as measured by quantitative OCTA, especially in those high myopes with lacquer cracks (LC) formation (15). Liu et al. also identified the fact that the reduction of CT in longer eyes seems more identical than in contralateral eyes in anisomyopes (16). With a close correlation of the growth of the sclera, an identifiable alteration of CT has the potential to affect the photoreceptor layer by moving the retina backward or forward (17). In the last few years, a growing number of researchers have tended to choose choroidal vessel index (CVI), a newly developed indirect vascular parameter, to describe the proportion of both large and medium vascularity volume of the whole choroid (18). Liu et al. thought persistent near work could lead to a prolonged reducing CVI in myopic adults, along with a decreased perfusion of choriocapillaries in pediatric myopes (19). A similar decrease was exhibited by enhanced depth imaging optical coherence tomography (ED-OCT) in another study, focusing on the changing choroidal vasculature with intrinsic correlation to myopia and hyperopia.

Unlike CT, CVI seems to be free of the interferences of a dozen of confounding factors, including age, axial length, and spherical equivalent, according to some relevant studies, thus making it more precise in detecting the histological effect on choriovasculature (20). Whereas, there is a stream of research with inconsistent declarations. Some regarded axial length as an independent influencing factor of the decreased CVI, while this inner correlation wasn’t of significance now and often (21, 22). Spherical equivalent (SE) is another typical indicator of the severity of myopia, however whether it matters in CVI alteration is still controversial and unidentified (20, 23). These discrepancies may be attributed to a host of reasons, including the variance of individual choroidal vasculature or the potential impact of other confounding elements. Currently, the axial length/corneal radius ratio (AL/CR) was revealed to be more efficient in detecting the total variance of the refractive state of myopes, whereas few studies have focused on discussing its hidden relationship with chorioretinal vascular parameters, which may help explain the pathophysiological impacts on variable fundus vessels of myopia in early stages (24).

This study aimed to comprehensively explore the topographic distributions of retinal alterations of myopic patients by SS-OCTA, a newly developed three-dimensional quantification equipment. Besides analyzing distinctions of various ocular biomarkers among several separate layers of papillary and peripapillary compartments between myopia groups with differential axial lengths, we also fill in gaps in the unrevealed relationships between AL/CR ratio and retinal changes, making it a potential indicator and detector of myopic fundus microstructure, while help uncover the pathophysiological mechanism of early myopia progression.

## Method

### Subjects

This observational cross-sectional study included 100 myopic patients who were recruited from July 2022 to December 2022 in the refractive Clinic of the Ophthalmology Department of First Affiliated Hospital of Chongqing Medical University. All subjects were free of any systematic disease or autoimmune disease that may influence ocular microcirculation, had no history of any other ocular disease or taking drugs in the past 2 weeks, and had no abnormal fundus manifestations obtained by fundus examination under a slip lamp. Moreover, every participant had a best-corrected logMAR visual acuity of 0 (20/20) or better in each eye, with SS-OCTA image signal intensity greater than or equal to 8, and the intraocular pressure was within the normal range (10–21 mmHg). This study was approved by the ethics committee of the First Affiliated Hospital of Chongqing Medical University, following the ethical standards stated in the Declaration of Helsinki, and written informed consent was obtained from all participants.

The myopic subjects were divided into two groups according to their differential axial length (AL): the non-high myopia group, defined by AL > 24 mm but <26 mm, and the high myopia group, defined by AL ≥ 26 mm. All examinations were conducted by a trained operator between 8:30 AM and 11:30 AM with the intention of minimizing potential confounding impacts of diurnal rhythm.

### Ocular biometrical measurements

A stream of ocular examinations was taken on all participants included in this study. Best-corrected visual acuity (BCVA) was recorded with a standard logarithmic visual acuity chart, AL and CR were measured by Pentacam® AXL panoramic biometer (Oculus GmbH, Wetzlar, Germany), and IOP was measured by a piece of noncontact tonometry equipment (model NT-4000, Nidek Inc., Fremont, CA, USA). SER was calculated as the addition of the spherical power and half the magnitude of the cylinder power.

### Image acquisition and biometrical analysis

Images of retinal microstructure were acquired using SS-OCTA (VG200S; SVision Imaging, Henan, China). It utilized a swept-source vertical-cavity surface-emitting laser (VCSEL) with a wavelength of 1,050 nm, providing an axial resolution of 5 μm and a lateral resolution of 13 μm. The spectral width ranged from 990 nm to 1,100 nm, the A-line speed was 20 K A-scans per second, and the scan depth was 3 mm (25–27). Ocular biometrical parameters of retinal vessels were acquired with a scan mode of Angio 1,024 × 1,024 R4, covering an area of 6 mm × 6 mm centered on the papillary and peripapillary regions, where a signal intensity greater than or equal to 8 (on a scale of 10) degrees were collected.

The retina in the SS-OCTA images was divided into the superficial retinal layer and the deep retinal layer, covering the area between 5 μm beneath internal limiting membrane (ILM) and 10 μm beneath Bruch’s membrane. The superficial retinal layer was defined as the area ranging from 5 μm beneath internal limiting membrane (ILM) to one third of ganglion cell complex (GCC), which is composed of the RNFL layer and the superficial vascular plexus (SVP). The deep retinal layer referred to the inner retina, defined as the area ranging from 5 μm beneath internal limiting membrane (ILM) to 25 μm beneath inner nuclear layer/outer plexiform layer (INL/OPL), also the combination of the intermediate capillary plexus (ICP) and the deep capillary plexus (DCP). More specifically, we defined RNFL as the area ranging from 5 μm beneath ILM to the upper bound of GCL, SVP as the capillaries extending from the upper bound of GCL to one third of GCC, ICP as those extending from one third of GCC to the half of inner nuclear layer (INL layer), DCP as vessels covering the area between the half of INL and 25 μm beneath INL/OPL, and defined INL as the area between the lower bound of IPL and the upper bound of OPL. At last, we measured the biometrical characters of the combined retinal layers of GCL and IPL, which covering the area between the upper bound of RNFL and the lower bound of INL.

The choroid refers to the tissue ranging from the RPE–Bruch’s membrane complex to the choroid–sclera interface, composed of three separate layers. The choriocapillaris layer lies from the basal border of the RPE-Bruch’s membrane complex to 20 μm beneath it, whereas both the large and middle choroidal vascular layers are located 20 μm beneath Bruch’s membrane to the choroid-scleral junction (11, 28).

**Figure 1:**
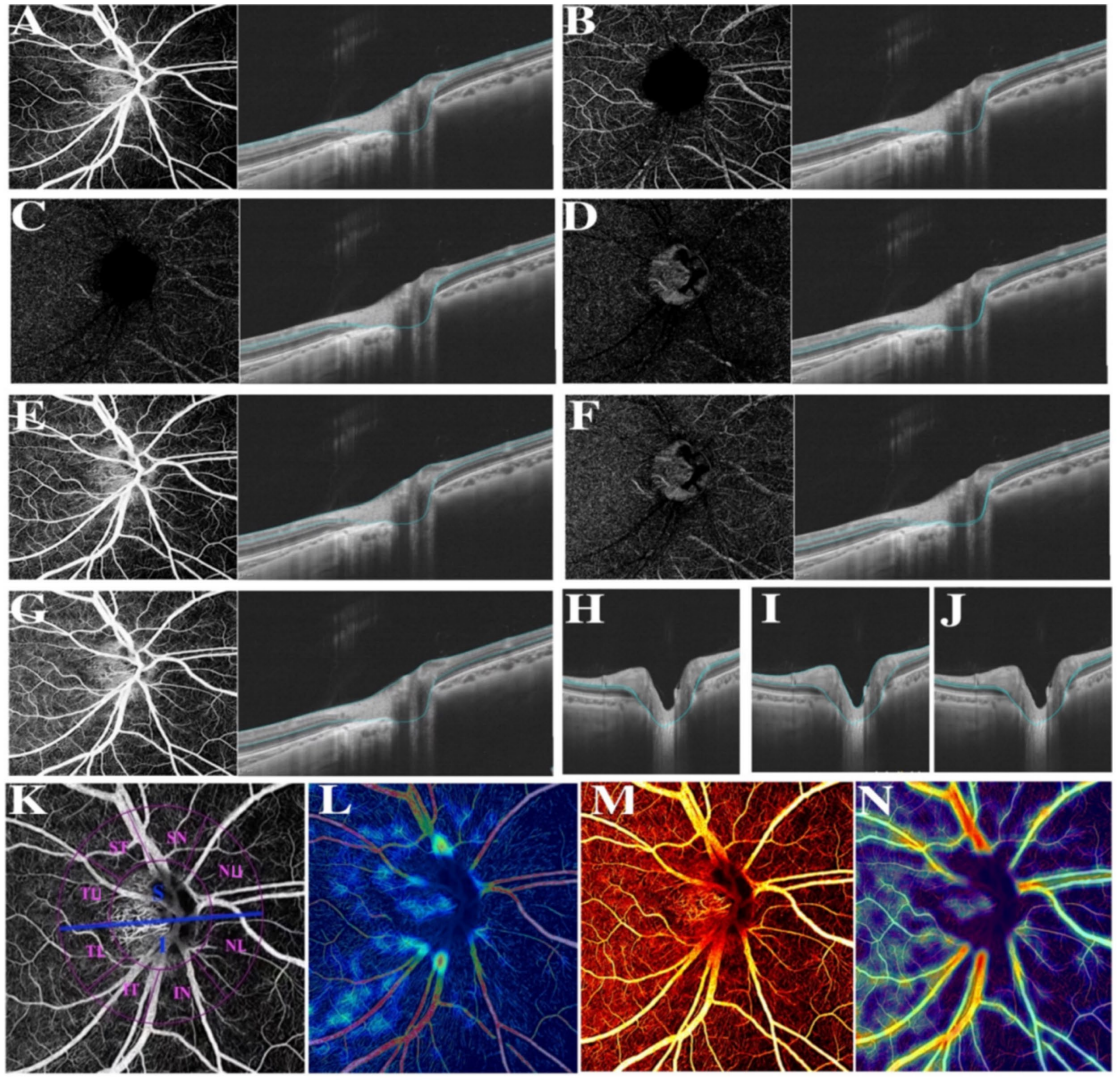
Various illustrations of retina. **(A)** RNFL. **(B)** The superficial vascular plexus (SVP). **(C)** The intermediate capillary plexus (ICP). **(D)** The deep capillary plexus (DCP). **(E)** The superficial retina refers to the vessels ranging from RNFL to SVP. **(F)** The deep retina refers to the vessels ranging from ICP to DCP. **(G)** The inner retina refers to the vessels ranging from 5 μm beneath ILM to 25 μm beneath INL/OPL. **(H)** The combined retinal layers of GCL and IPL refers to the area between the upper bound of RNFL and the lower bound of INL. **(I)** Ganglion cell complex (GCC). **(J)** INL refers to the area between the lower bound of IPL and the upper bound of OPL. **(K)** Different divisions of the optic disc. **(L)** Color coding of retinal small vessel density. **(M)** Color coding of retinal flow area. **(N)** Color coding of retinal vessel density.

Thereafter, compartmental analysis of a series of retinal biometrical parameters was taken between two myopic groups, including VD, the thickness and blood flow of separate retinal layers. Partially based on previous research, CVV was defined as the volume of the large and medium choroidal vessels, CVI was calculated as the ratio of the choroidal vascular volume to the total choroidal volume (27), VD, referred to the ratio of the projection of vessels in a certain fundus region divided by the total area (29). Further, the retinal thickness of various layers was also separately in estimation and comparison. According to the auto-protocol, compartmental analysis of the optic disc was performed based on the Garway-Heath sectorization. The optic nerve head (ONH) was divided into a 0–2 mm diameter area (papillary zone) and a 2–4 mm diameter area (peripapillary zone). The peripapillary region was further divided into superior (S) and inferior (I); simultaneously, the peripapillary annulus was subsequently divided into superonasal (SN), nasal upper (SU), nasal lower (NL), inferonasal (IN), inferotemporal (IT), temporal lower (TL), temporal upper (TU), supratemporal (ST) (Figure 1).

### Statistical analysis

Statistical analysis was performed using SPSS software (IBM SPSS Statistics version 27.0.1). Descriptive statistics covered variables including age, AL, and SE, assessed by ANOVA and expressed as the mean value ± standard deviation. Two sample-independent T-test was applied to identify intraocular differences in retinal parameters of various partitions between groups, after which Spearman correlation analysis was used to explore the potential correlation between fundus vascular variables and refractive parameters such as AL/CR ratio. In our study, a *p*-value of less than 0.05 indicated a statistically significant difference.

## Results

### General characteristics

All the ocular biometrical parameters were recorded from the right eyes of 100 myopes recruited in this study. A series of baseline characteristics are displayed in Table 1. Of these 54 males and 46 females participating in the study, the average age was 21.040 ± 4.151 years, and the mean AL, SE, IOP of cohort eyes were 25.878 ± 1.041 mm, −5.387 ± 2.693 D, 14.780 ± 2.592 mmHg, respectively. Significant distinctions were clearly exhibited when comparing AL, AL/CR ratio, and SE between two myopic groups, but not in comparisons of the age, gender, average k value, average CR or the differentiated pupil diameter.

**Table 1:** Demographic and ocular characteristics of participants in two myopic groups.

Parameters	Total	NHM (24 < AL < 26)	HM (AL ≥ 26)	*p* value
No. of eyes	100	59	41	
Age (years)	21.04 ± 4.15	21.27 ± 4.49	20.71 ± 3.64	0.491
Female (n)	46	29	17	0.453
SER (D)	−5.39 ± 2.69	−4.34 ± 2.07	−6.90 ± 2.79	<0.001
IOP (mmHg)	14.78 ± 2.59	14.90 ± 2.72	14.61 ± 2.41	0.587
AL (mm)	25.88 ± 1.04	25.18 ± 0.60	26.88 ± 0.64	<0.001
CR mean (mm)	8.18 ± 3.49	8.39 ± 4.54	7.89 ± 0.24	0.481
AL/CR ratio	3.28 ± 0.31	3.19 ± 0.36	3.41 ± 0.13	<0.001
K mean (mm)	43.11 ± 1.44	43.28 ± 1.55	42.86 ± 1.25	0.157
Pupil diameter (mm)	3.20 ± 0.24	3.20 ± 0.52	3.22 ± 0.66	0.874
ACD (mm)	3.23 ± 0.24	3.18 ± 0.20	3.30 ± 0.27	0.017
CCT (mm)	541.31 ± 35.52	541.66 ± 33.60	540.80 ± 38.54	0.906

### Compartmental analyses of retinal biometrical indicators

Aiming to further explore retinal microcirculation alterations, comparisons of a series of retinal biometrical indicators were therefore performed. VD and flow area of the whole retina and some separated retinal vessel layers were, respectively, estimated by compartmental analysis.

The superficial layer consisted of the retinal nerve fiber layer (RNFL) and SVP layer, and the deep layer was comprised of DCP and ICP layers. Obviously, non-highly myopes in group 1 exhibited a larger flow area of NU, NL sectors in the peripapillary area of the superficial layer and RNFL, while they also embraced a larger flow area in the papillary zone and TL sector of deep layer. Additionally, a remarkable decrease of flow area in SVP was found in the papillary area and the SN, IN sectors of the peripapillary area. Whereas, there were not such variances when turning to flow area of DCP or ICP, for only those of DCP in the palliary region and ICP in TL sector revealed statistical significance.

In the peripapillary region, VD of DCP, ICP and deep layer differed greatly between groups, exhibiting a giant decrease in both of the superior and inferior sectors in highly myopic eyes, VD of the RNFL and superficial layer in NU, NL, IN sectors was significantly differentiated, and VD of the retina was less in NU, IN, ST sectors in high myopes. To be more specific, NU, NL, and SN sectors had lower VD of DCP in high myopes, while NL, IN, ST, and SN had lower VD of ICP. Only a similar axial length-related decrease of SVP in IN sector was finally found.

For myopia patients with AL > 26 mm, the thickness of ganglion cell complex (GCC) in the optic disc and NU, NL, IN, SN sectors were significantly thinner in comparison, suggesting those of high myopia were generally thinner in the nasal peripapillary zone. A similar distinction was revealed in comparison of the thickness of the whole retina, which was also evidently thinner in high myopes. Additionally, they had a thinner RNFL thickness in NU, IN sectors, a thinner INL layer in the optic papilla, while lacking significant differences when analyzing the combined thickness of ganglion cell layer (GCL) and inner plexiform layer (IPL) in all measured areas.

**Figure 2:**
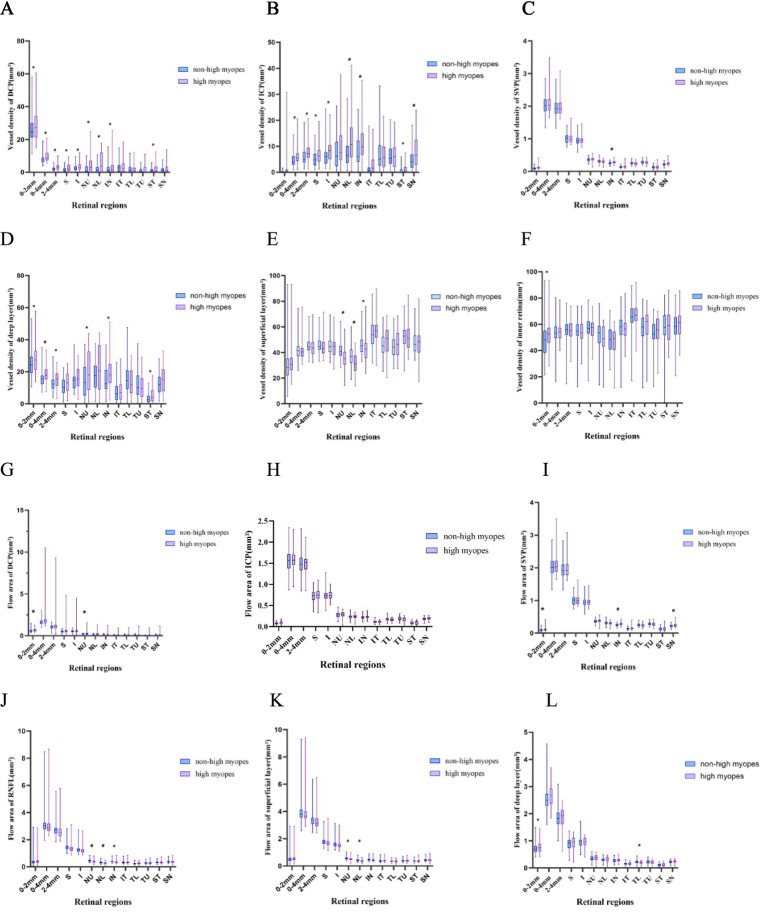
Box-plots showing the vessel density and flow area of different vessel layers. High myopes exhibited a lower vessel density, a lower small vessel density and a lower flow area, especially in the superficial layer and RNFL, along with a thinner superficial layer, RNFL and retina (*p* < 0.05). **(A–F)** Show the vessel density of DCP, ICP, SVP, superficial layer, deep layer and inner retina, respectively. **(G–L)** Represent the flow area of DCP, ICP, SVP, RNFL, superficial layer and deep layer, respectively. ^*^refers to a *p*-value less than 0.05, ^#^refers to a *p*-value less than 0.01, which reveal significant distinction between non-high myopes in group 1 and high myopes in group 2.

**Figure 3:**
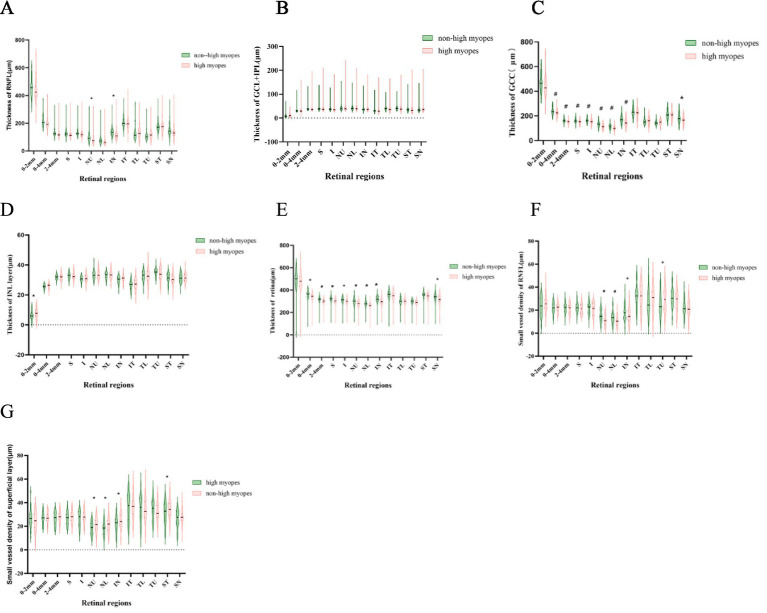
Violin-plots explicating the thickness of different retinal layers. A thinner superficial layer, RNFL and a thinner retina are explored in highly myopic participants (*p* < 0.05). **(A–E)** Represent the thickness of RNFL, SVP, superficial layer, INL layer and the whole retina. **(F,G)** Show the small vessel density of RNFL and superficial layer. ^*^refers to a *p*-value less than 0.05, ^#^refers to a *p*-value less than 0.01, which reveal significant distinction between non-high myopes in group 1 and high myopes in group 2.

Further, we individually compared the small vessel density of RNFL and the superficial layer among all compartments, as recorded in Figure 2. Myopes with shorter eyes in group 1 exhibited a larger small vessel density of RNFL in NU, NL, IN, TU sectors, and a larger small vessel density of the superficial layer in in NU, NL, IN, ST sectors (Figure 3).

### Correlation between AL/CR ratio and retinal parameters in the papillary and peripapillary regions

Spearman analysis was performed, respectively, to explore potential relationships among a stream of retinal biometrical indicators and AL/CR ratio in different myopic groups, as shown in Tables 2, 3. In those myopes with shorter axial lengths in group 1, the thickness of the whole retina, RNFL and GCC in the papillary and peripapillary zone were negatively correlated with AL/CR ratio, so was the combined thickness of GCL and IPL in the papilla. However, there was no significant relationship found between these indicators and AL/CR ratio in highly myopic eyes.

**Table 2:** Correlations of the interocular differences in AL/CR ratio with retinal parameters in the papillary region.

	NHM (24 < AL < 26)		HM (AL ≥ 26)	
Parameters	*r*	*p*	*r*	*p*
Thickness (μm)				
RNFL	−0.373	0.004^#^	−0.143	0.149
SVP	−0.346	0.007^#^	0.197	0.217
Superficial layer	−0.372	0.004^#^	−0.133	0.406
Retina	−0.328	0.011^*^	−0.049	0.760
Vessel density				
RNFL	0.354	0.006^#^	0.317	0.043^*^
SVP	0.362	0.005^#^	0.233	0.143
ICP	0.082	0.535	0.316	0.044^*^
DCP	0.271	0.038^*^	0.225	0.157
Superficial	0.350	0.007^#^	0.339	0.937
Deep	0.283	0.030^*^	0.192	0.230
Inner retina	0.430	<0.001^#^	0.323	0.039^*^
Flow area				
Superficial	0.259	0.048^*^	0.199	0.212
Deep	0.316	0.015^*^	0.184	0.248
Inner retina	0.368	0.004^#^	0.312	0.047^*^
Outer retina	0.263	0.044^*^	0.103	0.520
Retina	0.440	<0.001^#^	0.291	0.065

* refers to a *p*-value less than 0.05, reveals statistical significance.

# refers to a *p*-value less than 0.01.

All were calculated by Spearman correlation analysis.

**Table 3:** Correlations of the interocular differences in AL/CR ratio with retinal parameters in the peripapillary region.

	NHM (24 < AL < 26)		HM (AL ≥ 26)	
Parameters	*r*	*p*	*r*	*p*
Thickness (μm)				
RNFL	−0.387	0.002^#^	−0.229	0.149
SVP	0.047	0.721	−0.182	0.255
Superficial layer	−0.404	0.002^#^	−0.247	0.120
Retina	−0.263	0.044^*^	−0.232	0.145
Vessel density				
RNFL	0.032	0.809	0.249	0.117
SVP	0.220	0.094	0.149	0.354
ICP	0.158	0.232	0.349	0.025^*^
DCP	−0.195	0.139	0.152	0.342
Superficial	0.032	0.813	0.205	0.198
Deep	−0.007	0.959	0.021	0.898
Inner retina	−0.047	0.721	0.159	0.321
Flow area				
Superficial	0.075	0.573	0.085	0.596
Deep	−0.103	0.436	−0.036	0.822
Inner retina	0.368	0.004	0.124	0.439
Outer retina	0.058	0.663	0.112	0.487
Retina	0.033	0.803	0.178	0.267

* refers to a *p*-value less than 0.05, reveals statistical significance.

# refers to a *p*-value less than 0.01.

All were calculated by Spearman correlation analysis.

The correlations between AL/CR ratio and VD of the inner retina, superficial layers, deep layers and four single vessel layers were also analyzed individually. In the papillary region, for non-highly myopic patients, the vessel densities of all vessel layers were revealed to be positively correlated to AL/CR ratio except ICP, while no significant relationships were found in the peripapillary zone. However, for high myopic patients, the hidden correlations were only evident when comparing the VD of RNFL, ICP and inner retina with AL/CR ratio. Additionally, in the papillary region, the flow area of superficial layers, deep layers, inner retina, outer retina and the whole retina was all closely positively correlated to AL/CR ratio in non-high myopes in group 1, while for those high myopes in group 2, only the flow area of inner retina was positively correlated.

### Multiple regression analysis between characteristics of RNFL and ocular parameters in the papillary and peripapillary regions

Multiple regression analysis was performed individually on a series of ocular and demographic parameters to further explore their correlation to some retinal biometric indicators, as shown in Table 4. Accordingly, parameters such as sex (*β* = −0.261, *p* < 0.05), SE (*β* = 0.340, *p* = 0.01) and ACD (*β* = −0.350, *p* = 0.02) are significant factors affecting RNFL thickness in non-high myopic eyes, while sex (*β* = 0.254, *p* < 0.05) and ACD (*β* = 0.303, *p* = 0.02) are also identified to be significantly related to the vessel density of RNFL. However, correlations with parameters including CCT, AL and corneal radius are not statistically significant (*p* > 0.05), and no significant factor was identified in the high myopic group either.

**Table 4:** Multiple regression analysis on correlations between characteristics of RNFL and various ocular parameters in non-high myopic eyes.

Parameters	*β*	*p*
Thickness of RNFL (μm)		
Sex	−0.298	032^*^
Age	117	423
SE	369	0.010^#^
AL	226	0.148
Mean CR	−0.025	0.840
ACD	−0.342	023^*^
Vessel density of RNFL		
Sex	0.254	0.047^*^
Age	0.225	0.099
SE	−0.360	0.006^#^
AL	−0.123	0.390
Mean CR	−0.056	0.629
ACD	303	0.028^*^
Flow area of RNFL		
Sex	0.168	0.237
Age	307	0.046^*^
SE	−0.106	0.459
AL	015	0.925
Mean CR	−0.089	490
ACD	250	104

* refers to a *p*-value less than 0.05, reveals statistical significance.

# refers to a *p*-value less than 0.01.

## Discussion

In this study, we specifically analyzed the potential intraocular differences between myopic groups with different axial lengths, including a series of retinal biometrical parameters. For high myopes with an axial length of more than 26 millimeters, they exhibited a lower VD, flow area and small vessel density, especially in the superficial layer and RNFL, along with a thinner superficial layer, RNFL and retina. Further, the regional distribution characteristics of peripapillary regions were explored by compartmental analysis, suggesting that the retinal capillary network in the nasal peripapillary was more easily affected during the axial length elongation in high myopes. However, we also analysed the differences in CVV and CVI across two myopic groups, whereas the statistical findings of comparisons were not significant and seemed controversial, so we didn’t bring it up for discussion. By conducting correlation analysis, we finally explored the potential relationships between these ocular parameters and AL/CR ratio, suggesting that in non-high myopic eyes, the flow area and VD of the superficial and deep layers were significantly correlated with AL/CR ratio, so as the vessel density. Whereas, for highly myopic patients, only the VD of RNFL, ICP and inner retina in the papillary area and flow area of the inner retina were exhibited closely related to AL/CR ratio. Additionally, for myopes with shorter eyes, significant correlations were explicited after comparing AL/CR ratio with the thickness of the superficial layer, RNFL, and retina in the papillary and parapapillary zone, respectively. For those with AL > 26 mm, on the contrary, no correlations has been identified between AL/CR ratio and the thickness of various vessel layers.

The results in this compartmental analysis indicated that the characteristics and distribution of retinal capillaries of RNFL and superficial layer are highly differentiated by the degree of myopia, while the nasal peripapillary regions seems more easily to be impacted than the others, given that their vessel densities are exhibited generally lower. This is in accordance with some previous studies, which also indicated these distinctions and vascular changes could be seen among different myopic patients in sight of their perfusion of superficial layers, RNFL and other layers. How could these vascular changes occur during the process of myopia progression? The scleral remodeling theory is probably a reliable explanation. The Scleral, a highly-organized fibrous outer layer containing collagen and elastic fiber, serves as an ocular stabilizer to maintain biological structures by providing a stable base for the contractions of the ciliary muscle. Lately, visual signaling on the retina has been proven to be an imperative reflection during scleral remodeling, suggesting that changes in retinal vascular variables may be closely related to the degree and progress of myopia (8). A recent review shows that the excessive stretching of the retina secondary to axial elongating and scleral remodeling is one of the most vital pathological manifestation (30). Due to a temporal shift of axial elongation in myopic eyes, especially high myopia, a gradual stretching of retinal ganglion cell axons combining with a potential enlargement of Bruch membrane will possibly occur, ultimately resulting in a number of retinal structural abnormalities and variousness, including pan-retinal area thinning (31). Besides, in another former clinical experiment, the peripapillary thickness of the experimental high myopia model of juvenile tree shrews was quantified under 3D reconstructed segmentations, providing a view that the peripapillary tissues are prone to become significantly and heterogeneously thinner at an early stage after experiencing a process of experimental high myopia (32). This observation may make the point for increasing the risk for pathological optic nerve head remodeling. Vice versa, pathological influence on the retina also has the ability to impact myopic sclera. In an avian model of myopia, Jones identified that the biological properties of scleral structure could be changed by retinal image degradation through the mechanism of inducing eye growth and increasing a variety of related scleral proteinases (33).

Although the reason why histological changes in our study seem more significant in nasal peripapillary sectors is still unclear, there are some possible hypotheses that could be taken into account. First, anatomically, researchers certified there were less tortuosity and fewer vessel branches in nasal quadrants compared to the temporal side (34). This discrepancy of the optic disc may lead to quicker and earlier arteriolar subdivisions in myopia fundus change, in accordance with our study that nasal capillaries in the peripapillary region are more easily affected by the progression of myopia. What’s more, dozens of structural changes have been identified so far during axial elongation in myopia progression. For instance, Lee (34) thought the position of the central vascular trunk at the posterior pole was nasally dragged, which may help explain the tendency of an early vascular alteration on the nasal side than the others. Another pathological manifestation of fundus vessel alteration during axial elongation could be the retinal vessel shift (RVS), whose occurrence was supported by a clinical experiment that was designed to compare the fundus photographies of retinal vessel distribution before and after 2 years of myopia progression (35). They also use the peripapillary retinal arteries angle (PRAA) to make quantitative descriptions of RVS from different periods, and proposed that the existence of RVS and narrowing of PRAA are both correlated with the excessive elongation of eyeballs, serving as a strong potential explanation of focal-specialized changes of myopic fundus. Furthermore, in nonpathological high myopia, optic nerve head abnormalities could be divided into 3 categories based on their different retinal epithelial structures (36). For example, peripapillary intrachoroidal cavitation (PICC) is a description of cavity-like changes occurring within choroidal layers in the peripapillary area, and also comes with retinal vasculature abnormalities. In some former research, its presence in nonpathological high myopia was possibly a consequence of axial elongation and correlated to age at some point (37). Chen thought the presence of PICC was negatively correlated with VD in the optic nerve head. Whereas, in the radial peripapillary capillaries layer, VD was negatively correlated to the peripapillary atrophy area (38). This could be another cause for distinctions among different peripapillary compartments.

Still, both similarities and differences are exhibited when comparisons are experienced between our study and some previous studies. For instance, we found superficial layer and RNFL had lower VD with the increase of myopia degree, and these pathological changes were more easily identified in the nasal peripapillary region. In accordance with our findings, Guo (39) indicated a thinner superficial vessel density in nasal, inferonasal parts of the peripapillary region, Lin (40) reported a gradually decreasing RNFL vessel density in the peripapillary region during axial elongation, which occurred earlier in superior, inferior and nasal sides, proved significantly correlated to peripapillary RNFL thickness. In contrast, Venkatesh (41) showed the pericentral superior and temporal RPC vessel densities were lower in myopia patients in a prospective cross-sectional study, whereas Fan (42) suggested there were no such distinctions found after comparing superficial vessel densities in different myopic groups, with no correlation to axial length neither. Additionally, a large amount of inconsistency was also evident in sight of various layer thicknesses among myopes. By using Cirrus HD OCT, Kang (43) found a decreasing RNFL thickness in most nasal peripapillary sectors during myopia progression, which is consistent with our study. However, Cheng (10) did not find any significant distinction of peripapillary retinal nerve fiber layer thickness distribution in pathological changes of axial elongation, which was exactly counter to our results.

Moreover, we had some significant findings when comparing the potential relationships between retinal vascular alterations and AL/CR in each myopic group, which was rarely focused on in previous research. Recently, a meta-analysis revealed that the higher myopic eyes were believed to have longer ALs, larger AL/CR ratio and lower mean CR value, especially in non-highly myopic patients (44). A variety of researchers put an eye on the impacts of these biometrical structural changes. Schuster (45) supposed a flatter corneal radius was linked to a thinner retina, while Gong (46) held the point that the degree and proportion of fundus tessellation could increase sharply following a larger CR value, which may be attributed to foveal retina thinning and choroidal thinning (47). These findings were not consistent. As a newly developed biometric indicator of refractive error, the AL/CR ratio has been proven to be more closely correlated with personal refractive status than either AL or CR alone, as it steadily increased during myopia progress before stabilizing (48, 49). In this study, we found that the hidden relationships between AL/CR ratio and retinal biometrical parameters were much more evident in non-high myopic eyes. For instance, it was statistically positively correlated to flow area and vessel density of all vascular layers in the papillary region, as Hashemi (50) found this may serve as a possible result of the persistence or increase in the vasculature.

Additionally, an increasing AL/CR value was also significantly correlated with a thinner retina in both the papillary and peripapillary regions, especially in the superficial layers. Contrarily, these microstructural changes were barely related to the AL/CR ratio of high myopes. Consequently, we proposed that the retinal microcirculation was more sensitive to the dynamic alteration of AL/CR during the early stages of myopia progression, particularly in the inner retina. However, as the degree of myopia increased, the value of AL/CR tended to be stabilized, therefore making the correlation unclear when axial length exceeded 26 mm.

The results of multiple regression analysis suggested that sex is a potential influencing factor of the thickness and VD of RNFL, while there were no such significances identified for age. ACD increased with elongation of axial length in myopic eyes (51), which was also proved to be associated with the vascular characteristics of RNFL, whereas AL was not found to be significantly correlated in either non-highly myopes or high myopes, respectively.

The study has several limitations, however. First, the participants included are most youngsters that lack representativeness, and the sample size is was also limited. Besides, several minor fundus changes may not be identified through our primary screening, including some abnormal microstructure in the optic disc that could have the potential to impact the final images on SS-OCT angiography. A dozen initial nonpathological morphologic changes of high myopia could also affect the comparisons, such as focal retinal degeneration and choroidal atrophy. Although SS-OCTA was originally performed as an advanced and proven detecting method for fundus vasculature, there are still some defects in its receiving images. Since motion artifacts are always inevitable in OCTA measurements, SS-OCTA is likewise susceptible to these artifacts, thus a series of more precise image processing algorithms are urgently needed. A relatively small vision field of 6 mm × 6 mm area and the possibility of algorithm failure on segmentation and lamination also contribute to the inherent design limitations. Finally, some confounding factors could not be excepted in our primary study design, such as IOP, cup-to-disc ratio (C/D) and anterior chamber depth (ACD), which probably influence our consequences.

Accordingly, it’s suggested to have further long-term clinical studies with a larger sample size as well as a larger age spectrum. Enlarging the scanning area also counts, considering the present technological dilemma that only the posterior pole retina is involved in the scanning field, resulting in its limited use of detecting a broader range of ocular fundus disease. Theoretically, accepting clearer vascular images of separate layers will be promising only if we accelerate the scanning speed to raise the resolution; therefore, a continually progressing detecting machine such as SS-OCTA is always of great help. Although the interference of inherent heterogeneity of retinal vasculature and potential structural changes of nonpathological myopia cannot be identified, we hope researchers could come up with a precise algorithm that could largely exclude or better quantify the anatomic effect on vascular structures in myopia progression. In short, considering the distinctions among present relative studies, it’ still controversial that under which mechanism or to which degree will myopia progress impact the fundus microcirculation and retinal structure. Consequently, further investigation is still requisite.

## Conclusion

In conclusion, this study demonstrates that the superficial flow in the nasal peripapillary region is more easily affected during the axial elongation, given that the vessel density and flow area of these sectors are evidently diminished in the more myopic eyes, and vascular changes of the superficial layer are found similar to those of RNFL. Additionally, we highlight a potential correlation between AL/CR ratio and several different vascular characteristics using SS-OCTA, which could serve as sensitive biometrical indicators of myopic damages on retinal microvasculature in early stages, while shedding light on the hidden mechanism of affected fundus alterations. However, more detailed pathological mechanisms of myopia progression still require further investigation.

## Data Availability

The original contributions presented in the study are included in the article/Supplementary material, further inquiries can be directed to the corresponding author.
